# Dysphagia aortica presenting as failure of performing transoesophageal echocardiogram

**DOI:** 10.1093/ehjcr/ytae007

**Published:** 2024-01-06

**Authors:** Hussain Khalid, Wei Wang, Mohammed Ruzieh

**Affiliations:** Division of Cardiovascular Medicine, University of Florida Health, 1600 SW Archer Road, PO Box 100288, Gainesville, FL 32610, USA; Division of Cardiovascular Medicine, University of Florida Health, 1600 SW Archer Road, PO Box 100288, Gainesville, FL 32610, USA; Division of Cardiovascular Medicine, University of Florida Health, 1600 SW Archer Road, PO Box 100288, Gainesville, FL 32610, USA

## Case description

**Figure 1:**
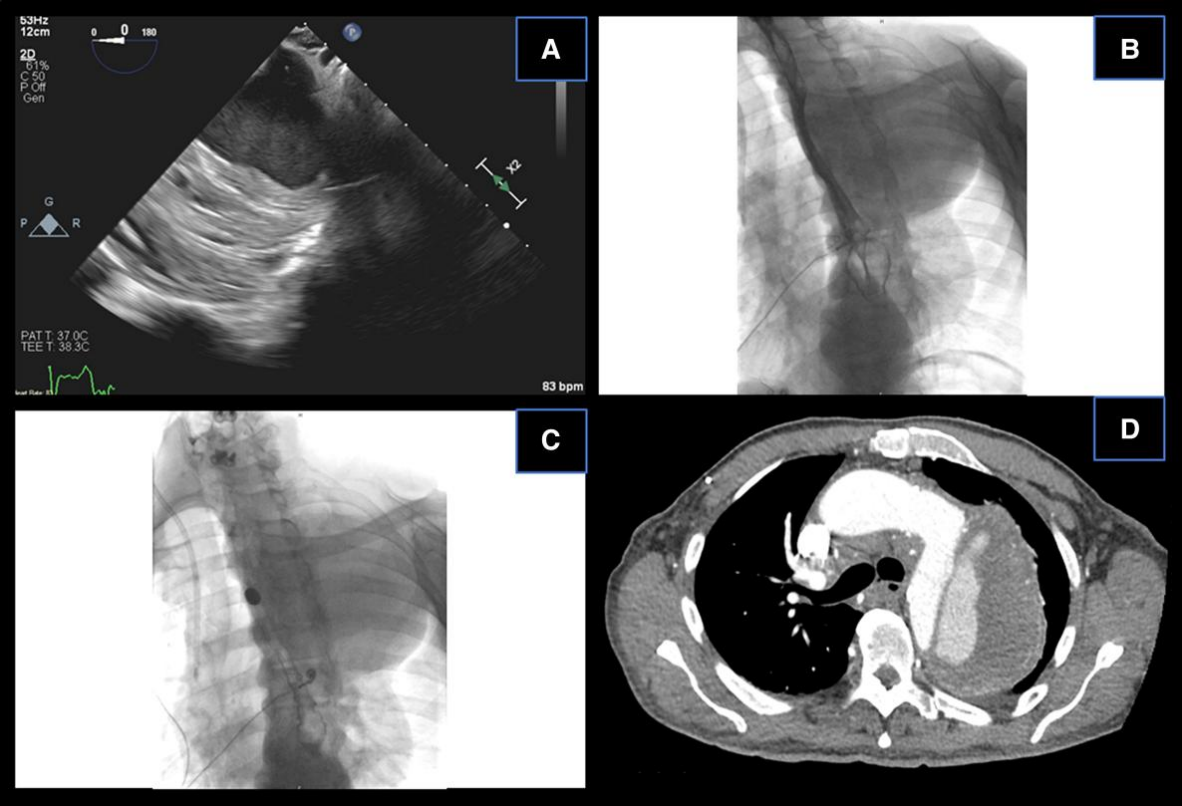
(*A*) A large 9.6 cm dissection of the aortic arch and proximal descending aorta with thrombosis of the false lumen as visualized on transoesophageal echocardiography. (*B*) Barium oesophagram demonstrating a large aortic aneurysm (dark silhouette in the upper left hemithorax) compressing the oesophagus at the T5 level. (*C*) Administration of a 13 mm barium tablet, trapped at the level T5 (level of oesophageal compression by the aortic aneurysm). (*D*) Computed tomography and angiography with a large DeBakey type III aortic dissection and partial thrombosis of the false lumen in close proximity to the oesophagus.

A 64-year-old man with history of DeBakey type III aortic dissection presented with altered mental status secondary to *Staphylococcus aureus* bactearemia. A transoesophageal echocardiography (TEE) was requested to rule out endocarditis. The patient’s spouse was consented for the procedure and indicated no history of dysphagia, odynophagia, oesophageal or gastric surgery, or other oesophageal or gastric pathology. During the procedure, the TEE probe could not be advanced further than the proximal oesophagus due to resistance. A large 9.6 cm dissection of the aortic arch with thrombosis of the false lumen was visualized. Oesophageal stricture secondary to external compression was suspected, and the procedure was aborted. Computed tomography and angiography (CTA) of the aorta confirmed a large aortic aneurysm with a prior dissection in close proximity to the oesophagus. Barium oesophagram demonstrated mass effect of the aortic dissection on the oesophagus at the T5 level (*Figure 1*). A 13 mm barium tablet was administered and became trapped at this level on the first swallow but passed after a second swallow with water (see Supplementary material online, *Videos S1–S3*). The patient later endorsed a longstanding history of dysphagia unbeknownst to his wife and was subsequently placed on a dysphagia diet and treated with a prolonged course of antibiotics and underwent thoracic endovascular aortic repair (TEVAR) after blood cultures cleared.

The term ‘dysphagia aortica’ was first described by Pape^1^ in 1932 as compression of the oesophagus from a dilated, torturous, or aneurysmal aorta.^2^ The incidence is rare, with only 70 patients reported in the literature over a 23-year period.^2^ This condition is typically treated with vascular surgery such as TEVAR, which may be combined with percutaneous endoscopic gastrostomy, oesophageal stenting, or oesophageal dilation.^2^ In patients with history of thoracic aortic pathology, there should be a high degree of suspicion for dysphagia aortica, especially in cases of difficult TEE probe advancement.

## Supplementary Material

ytae007_Supplementary_Data

## Data Availability

The data underlying this article are available in the article and in its online supplementary material.

## Supplementary material

**Consent:** Informed consent was obtained from the patient described in this case report. No identifying information or photographs were included in the manuscript. Standards according to Committee on Publication Ethics (COPE) and ICMJE recommendations for reporting were observed.

**Funding:** None declared.
