# Supplementary material for: Dysphagia aortica presenting as failure of performing transoesophageal echocardiogram
Source: Eur Heart J Case Rep. 2024 Jan 6;8(1):ytae007. doi: 10.1093/ehjcr/ytae007 (PMC10799656; doi:10.1093/ehjcr/ytae007)
Supplement: ytae007_Supplementary_Data [file ytae007_supplementary_data.zip › REVISED video legends Hussain TEE case report.docx]

**Video Legends**

**Video 1:** A large 9.6 cm dissection of the aortic arch and proximal descending aorta with partial thrombosis of the false lumen as visualized on TEE.

**Video 2:** Barium esophagram demonstrating a large aortic aneurysm (dark silhouette in the upper left hemithorax) compressing the esophagus at the level of T5. Administration of a 13mm barium tablet, trapped at level T5 (level of esophageal compression by the aortic aneurysm on the first swallow, with eventual passage of the barium tablet after a second swallow with water.

**Video 3:** CTA with a large DeBakey Type III aortic dissection and partial thrombosis of the false lumen in close proximity to the esophagus.
